# How personality traits shape susceptibility to mental distress in times of crisis: Evidence from the COVID-19 pandemic

**DOI:** 10.1371/journal.pone.0358371

**Published:** 2026-10-07

**Authors:** Marco Angrisani, Vincenzo Atella, Wändi Bruine de Bruin, Joanna Kopinska

**Affiliations:** 1 University of Southern California, Center for Economic and Social Research and Department of Economics, Los Angeles, California, United States of America; 2 University of Rome - Tor Vergata, Department of Economics, Rome, Italy; 3 University of Southern California, Schaeffer Institute for Public Policy and Government Service, Sol Price School of Public Policy, Los Angeles, California, United States of America; 4 University of Rome - La Sapienza, Department of Economics, Roma, Italy; Instituto Superior de Contabilidade e Administracao de Lisboa - Instituto Politecnico de Lisboa (ISCAL-IPL), PORTUGAL

## Abstract

Empirical evidence indicates that societal crises and adverse conditions can negatively impact population mental health. The Big Five personality traits – neuroticism, extraversion, openness, agreeableness, and conscientiousness –significantly influence stress responses. In this article, we examine how personality traits were associated with mental distress during and in the aftermath of the COVID-19 pandemic. Based on data from the Understanding America Study, we find that, since the pandemic began, individuals with high levels of neuroticism were more likely to experience mental distress. By contrast, the remaining personality traits exhibited weaker, inconsistent, or statistically insignificant associations with mental health trajectories. Further analyses indicate that the heightened vulnerability among individuals high in neuroticism was accompanied by elevated risk perceptions, differential adoption of protective behaviors, and less adaptive coping patterns. Taken together, these findings suggest that personality-linked differences, particularly driven by the level of neuroticism, help explain heterogeneity in mental health responses to large-scale societal stressors. Our findings point to the relevance of personality traits (particularly neuroticism) for identifying individuals who may benefit from targeted mental health support during future crises.

## 1. Introduction

Major societal stressors – whether sudden natural disasters, prolonged economic downturns, or global health emergencies – often lead to spikes in anxiety, depression, and loneliness, yet psychological responses vary widely. The COVID‑19 pandemic, as a recent example of a global crisis, exemplifies this pattern. Early evidence documented sharp rises in mental distress during lockdowns and uncertainty about future states [1,2]. A meta-analysis by [3] estimated the global prevalence of stress at 29.6%, anxiety at 31.9%, and the prevalence of depression at 33.7% during the first months of the pandemic. Reporting these mental health outcomes has been linked to pre-existing vulnerabilities, social and economic factors, and individual differences in personality, underscoring the need to better understand the mechanisms underlying mental distress in times of crisis [4].

A large body of research has examined the evolution of mental health over a longer time span since the start of the pandemic. In a systematic review and meta-analysis of 65 longitudinal cohort studies [5], found that mental health symptoms peaked in March–April 2020 and then gradually returned toward pre-pandemic levels by mid-2020, although depressive and mood symptoms showed more limited improvements over time than anxiety measures. Drawing on 134 cohorts [6], reached a similar conclusion: average distress levels did not substantially increase at the population level, and the pandemic was not associated with a widespread “tsunami of mental illness,” though effects varied significantly by subgroup, with women and individuals with pre-existing mental health conditions showing larger and more persistent effects. Other systematic reviews and meta-analyses have confirmed this nuanced picture, indicating that, while initial spikes in mental distress were widespread, sustained mental distress was concentrated among specific at-risk subgroups rather than uniformly experienced across the population [7–9]. This body of evidence suggests that population-level averages may obscure important heterogeneity in pandemic-related mental health trajectories, and that identifying the individual-level factors driving sustained distress is a critical task.

The Big Five personality traits – neuroticism, extraversion, openness, agreeableness, and conscientiousness – represent one such set of individual-level factors. These traits are associated with mental distress, emotional regulation, and coping strategies [10,11] and have long been recognized as important predictors of psychological responses to stressful life events.

A growing number of longitudinal COVID‑19 studies have examined how the Big Five personality traits relate to mental health during the pandemic. Using data from the UK Household Longitudinal Study with seven waves of COVID‑19 surveys (April 2020–January 2021) compared to a pre-pandemic baseline (2017–2019) [12], found that agreeableness protected against deteriorating mental health, while openness and extraversion were risk factors for deteriorating mental health. [13] showed that neuroticism was associated with stronger psychological and behavioral responses to the pandemic, including greater anxiety and more avoidance behaviors. [14] reported that neuroticism was the strongest predictor of declines in subjective and psychological well-being during COVID‑19, while conscientiousness had a protective effect. Taken together, these studies identify neuroticism as the most consistent risk factor for pandemic-related mental distress, while also highlighting the protective role of traits such as conscientiousness and agreeableness.

Alongside this longitudinal evidence, a parallel literature has investigated how personality traits are associated with risk perceptions, adoption of protective behaviors, and coping strategies. Individuals scoring high on neuroticism reported greater rumination and perceived higher infection and mortality risks during the pandemic [15], consistent with earlier evidence linking neuroticism to heightened risk perception and worry [16]. Conscientiousness, marked by self-discipline and goal-directed behavior, is linked to better stress management and healthier lifestyles, thus promoting overall well-being [17]. Individuals high in conscientiousness showed greater adherence to public health guidelines and protective behaviors during COVID-19, which likely mitigated pandemic-related mental distress [18]. Agreeableness, emphasizing cooperation and social harmony, fosters emotional-support networks that buffer against mental distress [19]; consistent with broader evidence linking agreeableness to prosocial and cooperative behavior [20], highly agreeable individuals during the pandemic engaged more in prosocial behaviors, including community support and compliance with public health measures. Extraversion, which tends to include sociability and positive emotions, is often linked to greater resilience but also carries risk when social interaction is restricted; individuals high in extraversion experienced pronounced strain under forced isolation [14,21,22]. Finally, openness to experience, which reflects curiosity and adaptability, correlates with cognitive flexibility and smoother psychological adjustment to change [23], though [13] found that individuals high in openness also exhibited stronger emotional responses to the pandemic. However, the mechanisms linking personality to mental distress through risk perception, protective behaviors, and coping strategies remain poorly integrated in a single analytical framework [24].

An additional dimension that warrants attention is age-related heterogeneity in both personality traits and mental health trajectories during the pandemic. Research on the Big Five has consistently shown that personality traits may vary across the lifespan: neuroticism and extraversion tend to decline with age, while agreeableness and conscientiousness tend to increase with age [25]. Evidence from the COVID-19 period indicates that personality traits, especially neuroticism, remained largely stable over the course of the pandemic [26]. The mental health consequences of COVID‑19 have varied across age groups, with younger adults aged 18–34 generally reporting more severe and persistent mental distress than their older counterparts [27]. It has been posited that these findings may reflect older adults’ better emotion regulation during the COVID-19 crisis [28,29]. However, direct evidence is missing about how relationships between personality traits and mental distress differ across age groups, and whether the mechanisms linking personality to pandemic-related distress operate differently at different stages of the life course.

This study examines the relationship between the Big Five personality traits and mental distress during high-stress societal events, utilizing the COVID‑19 pandemic as a case study. To this end, we rely on longitudinal, nationally representative data from the Understanding America Study (UAS). Managed by the University of Southern California, the UAS is a probability-based Internet panel that recruits participants nationwide via address-based sampling and supplies Internet access and devices to maximize representativeness. We integrate the Understanding Coronavirus in America Survey [30] with additional UAS surveys to obtain pre- and post-pandemic mental health measures, yielding a dataset spanning 2019−2024. This design enables us to track mental distress trajectories at both the population level and within personality-defined subgroups. Throughout this study, we conceptualize pandemic-related “uncertainty” as individuals’ subjective perceptions of health and economic risks associated with COVID-19. Empirically, we operationalize this construct using self-reported probabilities of infection, mortality, job loss, and financial hardship. These measures capture forward-looking assessments of adverse outcomes and represent a key mechanism through which personality traits may influence behavioral responses and mental distress. Additionally, we measure protective behaviors and potential coping strategies to shed light on other possible pathways through which personality traits predict mental distress in times of crisis.

Leveraging this unique source of data, we contribute to the literature on the relationship between personality traits and mental health in several ways. First, while the existing longitudinal evidence on personality and pandemic-related mental distress is growing [12–14], most studies cover a relatively short time window, typically confined to the first year of the pandemic. By contrast, our dataset enables us to examine trajectories of mental distress before, during, and well into the post-pandemic period. Second, our analysis is based on a large, population-representative panel, whereas much of the existing evidence, particularly from U.S.-based studies, relies on smaller or non-representative samples. Third, although prior work has separately documented personality-related variability in risk perceptions, protective behaviors, and coping strategies, our study examines these dimensions jointly within a single analytical framework to identify the pathways through which personality traits shape mental distress over the course of a major crisis. Fourth, we investigate how the relationship between personality traits and mental distress varies across three age groups, thereby addressing the gap in the literature on age-related heterogeneity in associations between personality traits and mental distress in times of crisis. Given that both personality traits and vulnerability to crisis-related distress differ across the lifespan [25], this age-stratified analysis provides novel insights into which subpopulations are most at risk and through which mechanisms.

## 2. Data and methods

### 2.1. Data

We analyze longitudinal data from the Understanding America Study (UAS), a nationally representative, probability-based Internet panel currently comprising approximately14,000 U.S. adults. The UAS was initiated in 2014 and has been managed by the Center for Economic and Social Research at the University of Southern California since [31,32]. Participants are recruited through address-based sampling from the U.S. Postal Service’s Computerized Delivery Sequence file, which covers nearly all U.S. households. Members complete surveys online; Internet connectivity and tablets are provided as needed to ensure representativeness.

Within the UAS, our primary data source is the Understanding Coronavirus in America Survey (UCAS, https://uasdata.usc.edu/page/Covid-19), a longitudinal survey designed to track attitudes and behaviors during the COVID-19 pandemic in the United States [30]. The UCAS was administered to the full UAS panel over 34 waves. Data collection began with a stand-alone survey fielded on March 10, 2020 (wave 1). From April 1, 2020 to February 2021, the survey was fielded biweekly (waves 2–24); from March through July 2021, it was fielded monthly (waves 25–29). Waves 30–34 were administered at irregular intervals in September–October 2021, February–March 2022, July–September 2022, November 2022–January 2023, and May–July 2023.

Measures of mental distress, described in more detail below, were collected in all UCAS waves, enabling the analysis of within-individual changes throughout the pandemic. Perceived risks of adverse outcomes associated with the coronavirus (e.g., infection, mortality, or job loss) were also elicited consistently across all waves. In contrast, measures of protective behaviors (e.g., mask wearing) and coping strategies that individuals adopted to mitigate pandemic-related stress were collected only through wave 30 (September–October 2021).

To construct a long-run panel of mental health outcomes, we merge UCAS data with additional UAS surveys. Pre-pandemic measures of mental distress are obtained from three UAS surveys (UAS 20, UAS 95, and UAS 185) completed between January 1, 2019 and February 1, 2020, which we define as the baseline period (wave 0). Post-pandemic outcomes are drawn from two later UAS surveys (UAS 592 and UAS 598) fielded in January–February 2024, which we define as wave 35. This structure allows us to observe mental health trajectories before, during, and several years after the pandemic.

The UAS also administers a set of core surveys that collect detailed background information, including family relationships, socioeconomic status, health, cognition, and individual traits (https://uasdata.usc.edu/page/Comprehensive±File). These surveys are conducted upon panel entry and subsequently every two years. The first wave in this core battery elicits measures of personality traits. In constructing our analytic dataset, we retrieved Big Five personality scores from wave UAS 121 of this core survey, fielded between January 2018 and August 2020.

Demographic characteristics were obtained from the My Household survey (https://uasdata.usc.edu/survey/My±Household), which is administered quarterly to all panel members and provides updated information on household composition and key socioeconomic variables.

By linking UCAS with additional UAS surveys, we constructed a longitudinal dataset containing repeated measures of mental distress spanning the pre-pandemic, pandemic, and post-pandemic periods; measures of risk perceptions, protective behaviors, and coping strategies during the pandemic; pre-pandemic personality traits; and detailed demographic information.

### 2.2. Measures

Mental distress was measured via the Patient Health Questionnaire-4 (PHQ-4). This brief self-reported scale, developed and validated by [33], consists of 4 items, 2 assessing depression symptoms (“feeling down, depressed or hopeless” and “little interest in doing things” and 2 assessing anxiety symptoms (“feeling nervous, anxious, or on edge” and “ not being able to stop or control worrying”) as experienced over the past 2 weeks [33]. Each item is answered on a 4-point Likert scale; hence, the PHQ-4 score ranges from 0 to 12, with low values corresponding to no mental distress symptoms and high values indicating more severe mental distress symptoms. In our analysis, we use both the PHQ-4 score as a continuous variable and a mental distress dichotomous indicator, defined as a PHQ-4 score≥6, which, in clinical settings, corresponds to moderate or severe symptoms of depression or anxiety [33]. The same PHQ-4 questionnaire was administered in all UCAS waves, as well as in the pre- and post-pandemic waves merged with the UCAS data. In our sample, the PHQ-4 exhibits excellent internal consistency, with a Cronbach’s alpha of 0.93.

In the UCAS, respondents reported their perceived probability (on a 0–100 scale) of contracting the coronavirus, being hospitalized, or dying from it within the next three months. They also reported the perceived probability of losing their job and running out of money due to the coronavirus over the same horizon (see S1 Appendix for the full questionnaire). In the analysis, we focused on three of these outcomes – perceived risk of death, job loss, and running out of money – as the remaining measures yield similar results.

To measure protective behaviors, respondents were asked: “Which of the following have you done in the last seven days to keep yourself safe from coronavirus? Only consider actions that you took or decisions that you made personally.” Reported behaviors included, among others, frequent hand washing or use of hand sanitizer, avoiding public spaces or gatherings, and wearing a mask or face covering. Responses were recorded as binary indicators (see S1 Appendix for the full list). We report results for hand washing, mask wearing, and avoidance of public spaces, as other behaviors displayed similar patterns.

The UCAS also elicited the frequency of potential coping activities by asking: “Out of the past 7 days, what is your best estimate of the number of days that you did each of the following activities?” Responses ranged from 0 to 7 days. Activities included relaxing, meditating, exercising, drinking alcohol, and using recreational drugs (see S1 Appendix). We present results for relaxing, exercising, and alcohol consumption, as findings for the remaining activities are qualitatively similar.

In the UAS, the Big Five personality traits are measured using the 44-item scale developed by [34]. Based on their responses, respondents receive a score ranging from 1 to 5 for each trait: neuroticism, extraversion, openness, agreeableness, and conscientiousness (the complete list of items is in S1 Appendix; the full scoring procedure is available at https://uasdata.usc.edu/survey/UAS±593). The internal consistency of the personality scales in our sample is high, with Cronbach’s alpha coefficients of 0.83 for neuroticism, 0.83 for extraversion, 0.78 for openness, 0.78 for agreeableness, and 0.79 for conscientiousness. In our analysis, we use personality measures elicited prior to the onset of the pandemic and treat them as time-invariant individual characteristics. This assumption is consistent with established evidence on the relative stability of personality traits in adulthood [35,36]. We also checked the plausibility of this assumption in UAS data. Over the period 2014–2025 in the UAS, there are 21,425 unique individuals with at least one personality trait measurement. The number of available measures per individual is between 1 and 5, with a median of 2. In this sample, personality traits exhibit high stability over time. Test-retest correlations between adjacent measures range from 0.70 to 0.82, indicating strong rank-order stability, whereby individuals largely maintain their relative positions in the trait distribution across waves. These estimates are fully consistent with the personality psychology literature, which typically reports test-retest correlations for the Big Five in the range of 0.70 to 0.85 in adult samples [35,36].

### 2.3. Statistical analysis

After listwise deletion of observations with missing outcome variables and/or covariates, the final analytic sample comprises 4,030 unique respondents and 106,668 respondent-wave observations across the 36 waves described above (wave 0 for the pre-pandemic period, waves 1–34 from the UCAS, and wave 35 for the post-pandemic period). Panel participation is high: 52% of respondents are observed in at least 32 waves, and 34% appear in all 36 waves. Only 11% are observed in fewer than 10 waves, with a minimum of two waves per individual. Individual characteristics and outcome variables at baseline are reported in Table A.1 in S1 Appendix. The results of attrition analysis are reported in Tables A.2 and A.3 in S1 Appendix. These results indicate that attrition in our sample is primarily driven by observable demographic characteristics rather than by baseline mental health or personality traits. This suggests that selective non-response is unlikely to materially bias our main findings, especially given that all analyses flexibly control for demographics.

Because UCAS waves were fielded at varying frequencies over time, and because pre- and post-pandemic measures were drawn from separate UAS surveys with different temporal distributions – spanning approximately one year in the pre-pandemic period and concentrated within two months in the post-pandemic period – we aggregate observations into broader periods corresponding to the main phases of the pandemic for the purposes of empirical analysis and graphical presentation.

Specifically, pre-pandemic observations from January to February 2020 are grouped into Period 0. Period 1 (March–June 2020) corresponds to the onset of the pandemic. Period 2 (July–October 2020) captures the first resurgence in COVID-19 cases, while Period 3 (November 2020–February 2021) reflects a subsequent resurgence. Period 4 (March 2021–January 2022) covers the initial phase following the vaccine rollout. Period 5 (February 2022–April 2023) corresponds to the final major resurgence prior to the official declaration of the end of the pandemic in May 2023. Finally, period 6 (May 2023–February 2024) represents the post-pandemic period. These seven aggregated periods form the time structure used throughout the analysis and in the figures.

Using this panel dataset and time structure, we performed the following sets of analyses. First, we examined how trajectories of mental distress evolved over time and how they varied by personality traits. To quantify these differences, we estimated regression models of mental distress – linear regression for the continuous PHQ-4 score and logistic regression for the mental distress dichotomous indicator (PHQ-4 ≥ 6) – on the set of aforementioned time period indicators (with period 0 as the reference), indicators for quartiles of a given personality trait (using the first quartile as the baseline), and their interactions. All models controlled for gender, race, age, education, marital status, household income, and the scores of the other four personality traits not interacted with time period indicators, and were estimated with standard errors clustered at the individual level.

From these models, we computed predicted PHQ-4 scores and predicted probabilities of mental distress for each personality trait quartile in each period, which we then plot in the figures below (the full set of estimated coefficients for the linear regressions with PHQ-4 score is reported in Tables A.4-A.8 in S1 Appendix). This regression-adjusted approach allows us to trace differences in mental health over time across levels of personality traits while holding observed individual characteristics constant. Accordingly, this method isolates the association between personality and mental distress net of compositional differences in other individual characteristics, thereby providing a clearer description of differential changes over time.

The interaction coefficients from the same models capture differential changes in mental distress between individuals in the higher quartiles (second through fourth) and those in the lowest quartile of a given trait for each period, relative to the pre-pandemic baseline. Statistically significant interaction terms indicate meaningful heterogeneity in trajectories of mental distress across personality levels over the different phases of the pandemic. In the figures included in this manuscript, we report the interaction coefficients for the fourth quartile to highlight contrasts between individuals with the highest and lowest levels of each trait; the full set of estimates is provided in S1 Appendix.

To explore heterogeneity across individuals at different stages in their lifetime, we replicate this analysis separately for three age groups: 18–39, 40–59, and 60 and older (we will follow [26] for the age class structure.). We substitute quartile indicators with a binary indicator for being above the median level of the personality trait to enhance statistical power.

Finally, we examined how personality traits were associated with risk perceptions and behavioral responses during the pandemic. For this purpose, we estimated regression models that include period indicators, a standardized measure (z-score) of a given personality trait, and their interactions. Risk perceptions (measured on a 0–100 scale) and the frequency of potential coping strategies (0–7 days) were analyzed using linear regressions, while the adoption of protective behaviors (binary indicators) was analyzed using logistic regressions. In all models, we included controls for demographics and the remaining personality traits. For protective behaviors, we estimated specifications both excluding and including risk perceptions as an additional control to assess the extent to which protective behavior was informed by perceived risk. From these models, we obtained period-specific marginal effects of a one–standard-deviation increase in the personality trait on each outcome. These estimates, which capture how the association between each personality trait and the respective outcomes evolves across the different phases of the pandemic, are reported in the figures below.

### 2.4. Ethics

The data collection was carried out within the Understanding America Study (UAS). Questionnaires were distributed via computer link between January 1^st^ 2019 and February 29^th^ 2024. Respondents provided informed consent to join the UAS and complete surveys online on a regular basis. Informed consent was obtained from all participants via computer link. All procedures involving human subjects were approved by the University of Southern California’s IRB (UP-14-00148) and BRANY (Biomedical Research Alliance of New York) IRB (22-030-1044).

## 3. Results

### 3.1. Population-level mental distress trajectories

At the population level, mental distress level, as measured by the continuous PHQ-4 score, and the prevalence of mental distress (PHQ-4 ≥ 6) rose sharply at the onset of the pandemic in March 2020, coinciding with nationwide lockdowns and unprecedented health and economic uncertainty (Figs 1 and 2). Although both measures declined after this initial surge, they did not return to pre-pandemic levels. Instead, average PHQ-4 scores and the probability of mental distress remained persistently elevated relative to 2019 throughout the subsequent phases of the pandemic and into the post-pandemic period. These patterns indicated a sustained shift upward in population-level mental health symptoms rather than a purely transitory shock.

**Fig 1:**
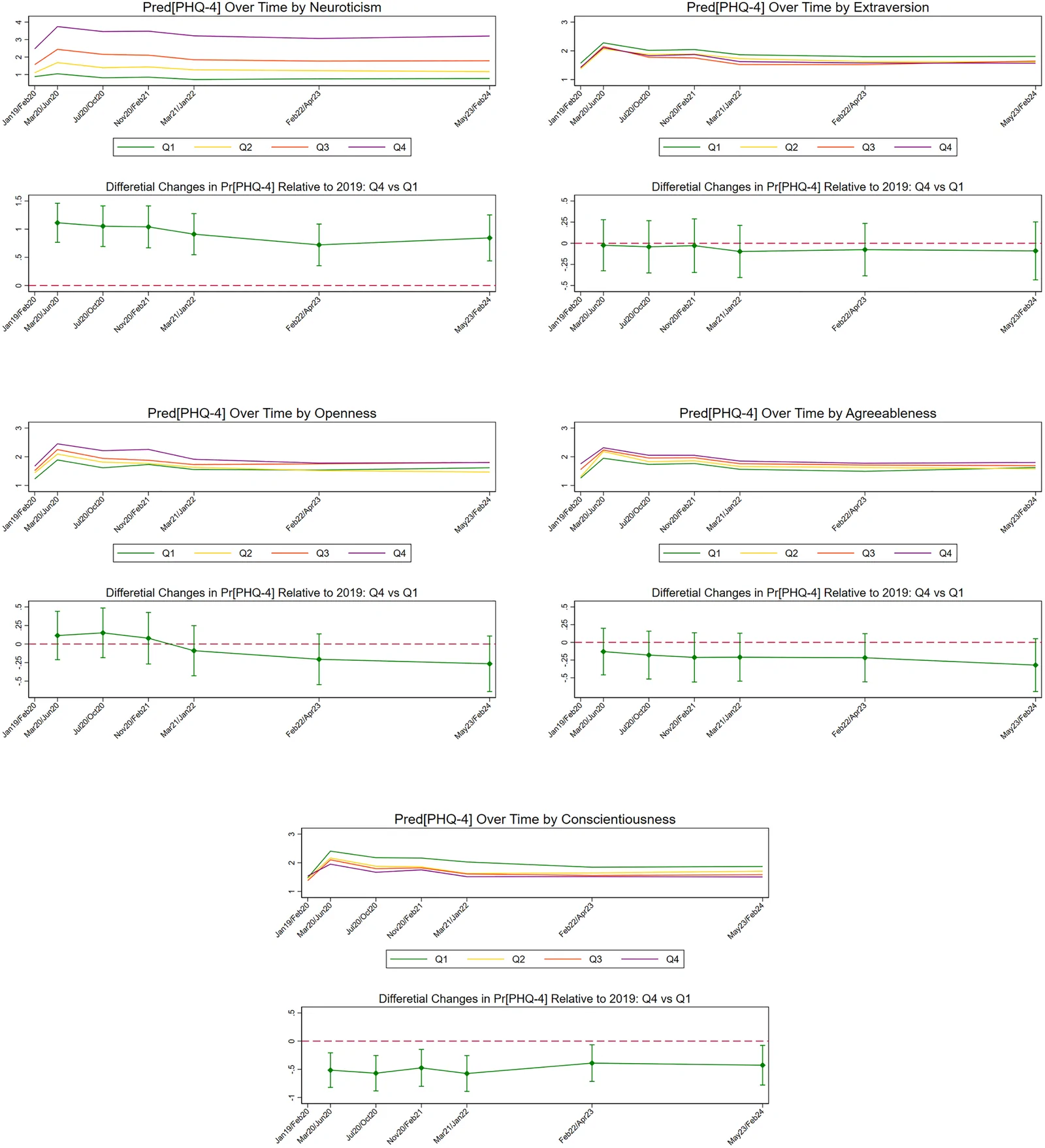
Predicted PHQ-4 score (Pred[PHQ-4]) over time by personality trait quartile. Note: In each panel, the top figure plots predicted PHQ-4 scores over time by quartiles of the corresponding personality trait. Predictions are obtained from linear regressions of PHQ-4 scores on period indicators, personality trait quartile indicators, and their interactions. All specifications control for gender, race, age, education, marital status, household income, and the scores of the remaining personality traits (as quartile indicators not interacted with time periods). The bottom figure in each panel plots the estimated interaction coefficients between the 4^th^-quartile indicator of the personality trait and the period indicators. These coefficients capture the differential change in PHQ-4 scores between individuals in the highest (4^th^ quartile) and lowest (1^st^ quartile) levels of the trait for each period, relative to the pre-pandemic baseline.

**Fig 2:**
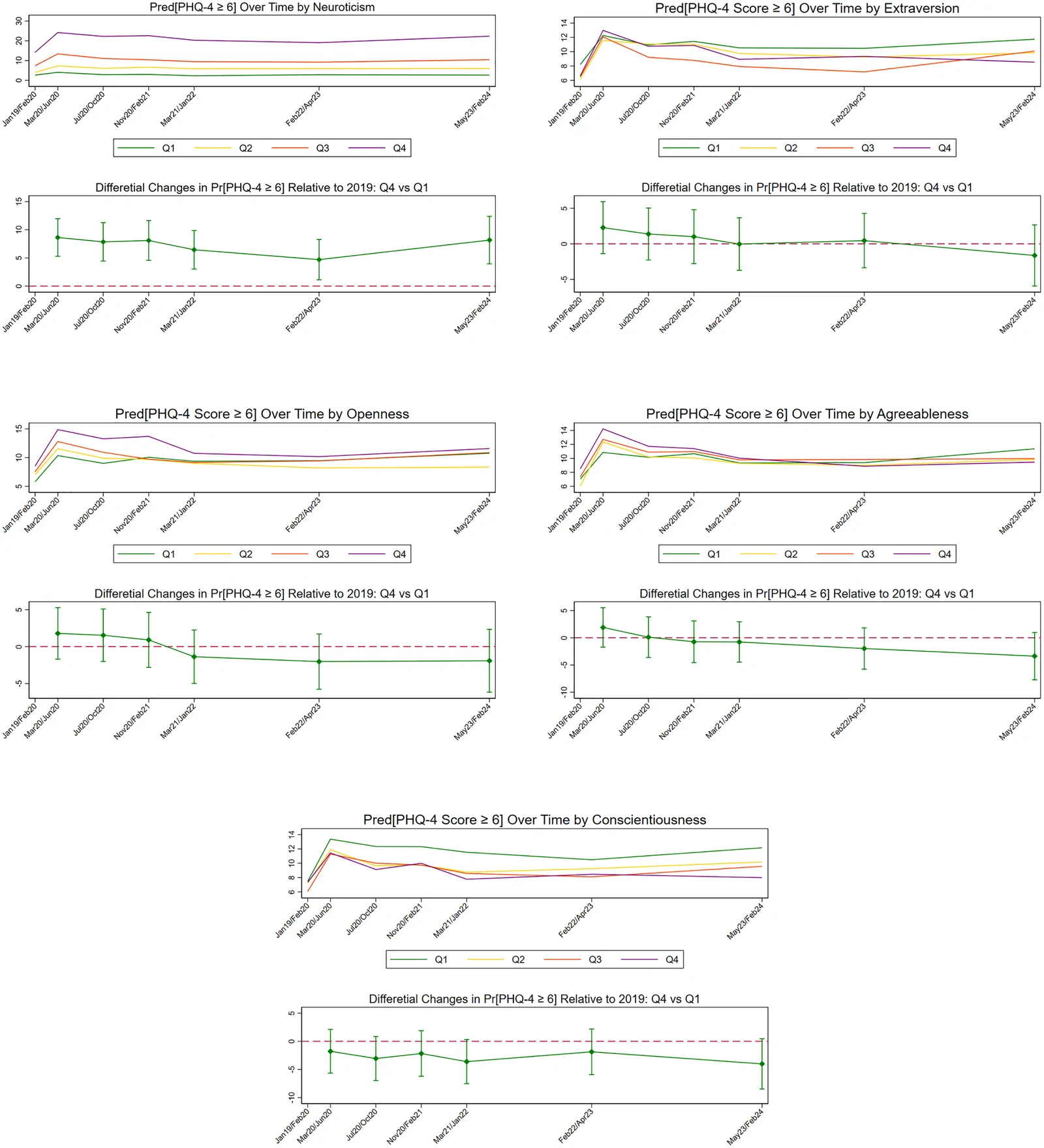
Predicted probability of mental distress (Pred[PHQ-4 ≥ 6]) over time by personality trait quartile. Note: In each panel, the top figure plots the predicted probability in percentage points of mental distress (PHQ-4 score ≥ 6) over time by quartiles of the corresponding personality trait. Predictions are obtained from logistic regressions of mental distress on period indicators, personality trait quartile indicators, and their interactions. All specifications include the same controls as in Fig 1. The bottom figure in each panel plots the the estimated differential probabilities of reporting mental distress between individuals in the highest (4^th^ quartile) and lowest (1^st^ quartile) levels of the trait for each period, relative to the pre-pandemic baseline.

### 3.2. Personality traits and mental distress trajectories

We document substantial and statistically significant heterogeneity in trajectories of mental distress by personality traits (Figs 1 and 2). Individuals with high levels of neuroticism experienced both the largest initial deterioration and the most persistent increase in mental health symptoms and distress. As shown in the top-left panel of Fig 1, relative to the pre-pandemic baseline, individuals in the highest quartile of neuroticism exhibited increases in PHQ-4 scores that were approximately 1.1 point larger than those observed among individuals in the lowest quartile during the early phases of the pandemic –equivalent to roughly a 70% increase relative to the pre-pandemic sample mean. Although this differential narrowed in later periods, it remained sizeable and statistically significant throughout the observation window.

A similar pattern emerged for the probability of mental distress (PHQ-4 ≥ 6), as shown in Fig 2. Relative to the pre-pandemic baseline, individuals in the top neuroticism quartile experienced increases in predicted mental distress probabilities that were approximately 11–20 percentage points larger than those in the bottom quartile throughout the pandemic period. These differential changes remained large and statistically significant over time, indicating a persistent association between higher neuroticism and greater vulnerability to sustained mental distress.

In contrast to neuroticism, the remaining personality traits exhibited weaker and less consistent heterogeneity in trajectories of mental distress. Extraversion showed virtually no systematic differences across quartiles. Openness displayed modest early heterogeneity, with individuals in the top quartile experiencing a somewhat larger deterioration relative to those in the bottom quartile; however, this pattern attenuated over time and was not statistically significant. Agreeableness showed a small protective pattern that remained statistically insignificant. Conscientiousness exhibited a modest protective association, with individuals in higher quartiles consistently exhibiting lower PHQ-4 scores; however, these differences were smaller than those observed for neuroticism and were generally not statistically significant when considering the binary measure for mental distress.

This discrepancy reflects the nonlinear relationship between continuous symptom severity and the binary distress threshold. While higher conscientiousness was associated with modest shifts in the PHQ-4 score, these changes were generally not large enough to move substantial numbers of individuals across the clinical cutoff for moderate-to-severe mental distress (PHQ-4 ≥ 6). As a result, effects remain detectable in the continuous mental distress measure but appear attenuated and often statistically insignificant in the binary outcome, where variation depends on transitions across the PHQ-4 ≥ 6 threshold rather than shifts within the distribution.

In contrast, the heterogeneity associated with neuroticism was substantially larger in magnitude and concentrated in the upper tail of the symptom distribution. Consequently, increases in symptoms among highly neurotic individuals translated into sizeable movements across the clinical cutoff, producing strong and statistically significant effects even in the binary distress specification.

### 3.3. Age-stratified analyses

Fig 3 examines whether the relationship between neuroticism and trajectories of mental distress varied across age groups. Increased mental distress during the pandemic was evident across young (18–39), middle-aged (40–59), and older (60+) adults alike. In all three groups, individuals with above-median neuroticism experienced a pronounced increase in both PHQ-4 scores and the predicted probability of mental distress at the onset of the pandemic, followed by partial recovery that remained above pre-pandemic levels. The magnitude of these differences was broadly comparable across age groups, suggesting that the vulnerability associated with higher neuroticism was not confined to a particular stage of the life course. The age-stratified results for the remaining personality traits are reported in Figures A.1–A.4 in S1 Appendix. For extraversion, openness, and agreeableness, we found little evidence of meaningful age heterogeneity in trajectories of mental distress, indicating that the largely null results observed in the full sample were not masking offsetting age-specific effects.

**Fig 3:**
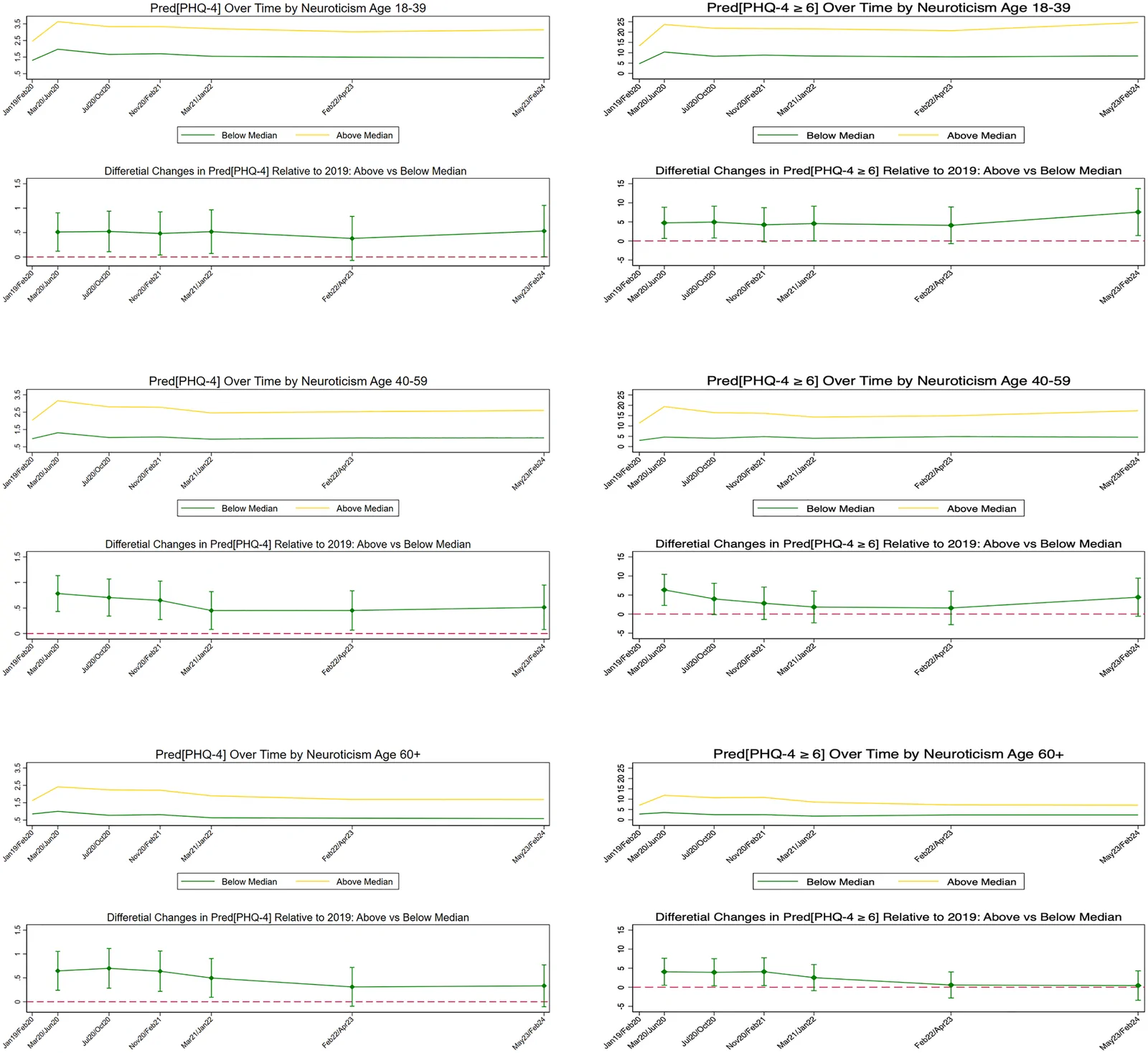
Predicted PHQ-4 score (Pred[PHQ-4]) and predicted probability of mental distress (Pred[PHQ-4 ≥ 6]) over time by neuroticism and age. Note: The figures in the left column refer to the predicetd PHQ-4 score; the reported results are obtained from linear regressions, as in Fig 1, estimated separately for each age group. The figures in the right column refer to the predicted probability of mental distress (PHQ-4 score ≥ 6); the reported results are obtained from logistic regressions, as in Fig 2, estimated separately for each age group. Relative to the linear and logistic regressions in Figs 1 and 2, neuroticism quartile indicators are replaced with a binary indicator for being above the median level of neuroticism in order to enhance statistical power.

In contrast, the age-stratified patterns for conscientiousness suggested that the protective association was more pronounced among older adults. Among individuals aged 60 and above, those with above-median conscientiousness consistently exhibited lower PHQ-4 scores and a lower probability of mental distress throughout the pandemic, whereas differences were substantially weaker and generally statistically insignificant among younger and middle-aged adults.

### 3.4. Mechanisms: Risk perceptions, protective behaviors, and coping strategies

The pandemic-related increase in mental distress observed among individuals with higher levels of neuroticism was accompanied by distinct patterns in risk perceptions, protective behaviors, and potential coping strategies over the course of the pandemic. Panel (a) of Fig 4 examines the association between neuroticism and perceived risks. A one–standard deviation increase in neuroticism was associated with significantly higher perceived probabilities of death, job loss, and financial hardship due to COVID-19. These associations peaked during periods of elevated infections and declined only gradually through late 2022 and thereafter.

**Fig 4:**
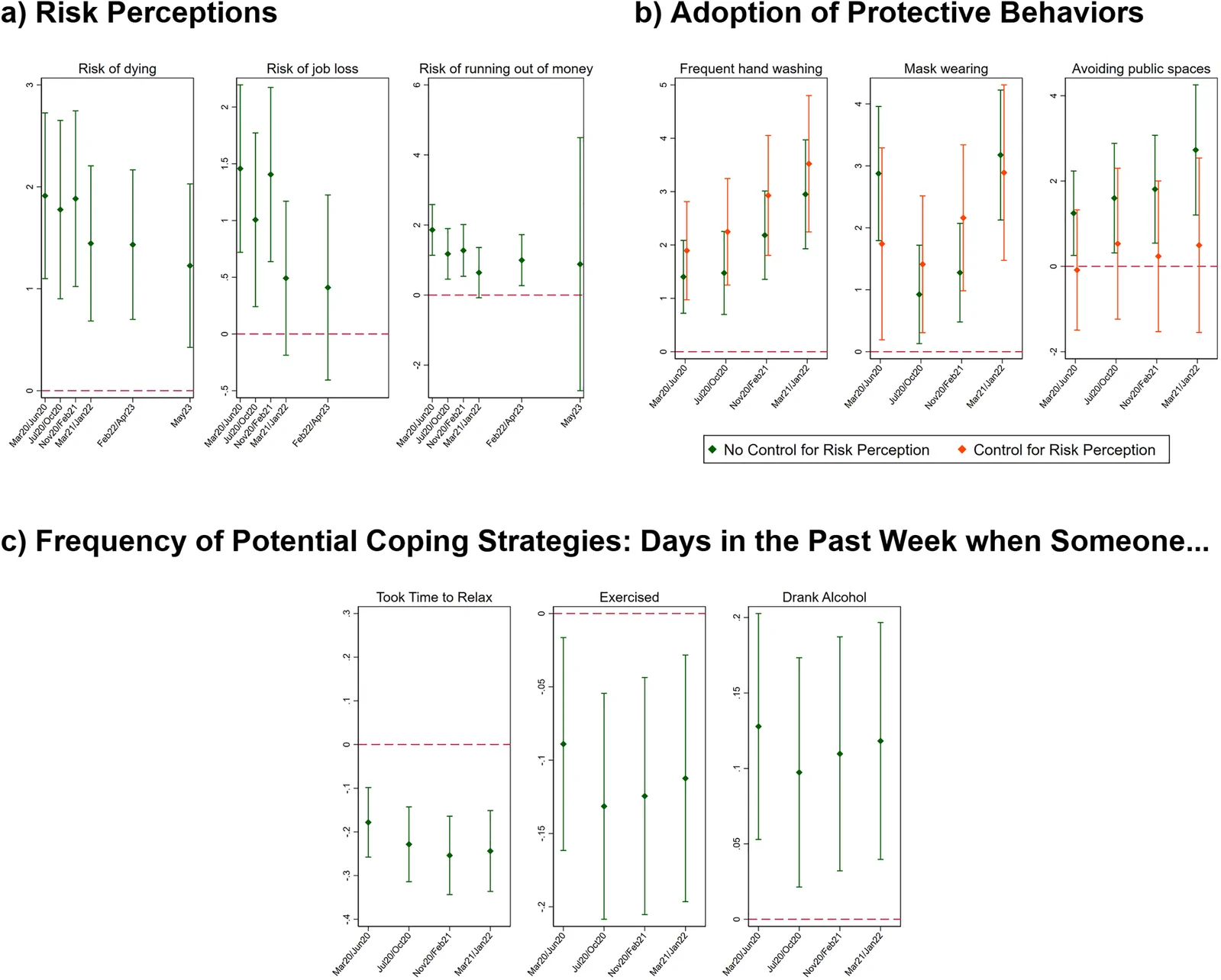
Marginal effects of neuroticism on risk perceptions, adoption of protective behaviors, and frequency of potential coping strategies. Panel a) displays the estimated marginal effects of neuroticism on risk perceptions for each period, based on OLS regressions of risk perceptions (0–100%) on period indicators, standardized neuroticism, and their interaction. Each point represents the change in predicted risk perception (in percentage points) associated with a one–standard-deviation increase in neuroticism. Panel b) displays the estimated marginal effects of neuroticism on the likelihood of adopting a protective behavior for each period, based on logistic regressions of an adoption indicator on period indicators, standardized neuroticism, and their interaction. Each point represents the change in the predicted probability (in percentage points) of adoption associated with a one–standard-deviation increase in neuroticism. Green estimates exclude risk perceptions as a control; orange estimates condition on risk perceptions. Panel c) displays the estimated marginal effects of neuroticism on the frequency of a potential coping strategy (days 0–7) for each period, based on OLS regressions including period indicators, standardized neuroticism, and their interaction. All regressions control for gender, race, age, education, marital status, household income, and the other personality traits. Horizontal bars denote 95% confidence intervals based on standard errors clustered at the individual level.

Panel (b) documents a strong positive relationship between neuroticism and the adoption of protective behaviors, such as frequent handwashing, mask wearing, and avoidance of public spaces. The magnitude of this association increased steadily through 2021 (behavioral data are available only through the second COVID surge in fall 2021). For handwashing and mask wearing, the estimated effects were similar with and without controlling for individual risk perceptions, suggesting that these behaviors were not solely driven by heightened perceived risk. By contrast, the association between neuroticism and avoidance of public spaces became statistically insignificant once risk perceptions were included in the model, indicating that this behavior operated largely through perceived risk channels.

Panel (c) shows that higher neuroticism was also associated with distinct coping patterns: individuals with higher neuroticism reported lower frequencies of relaxation and physical activity, but higher levels of alcohol consumption. Taken together, these findings pointed to multiple, complementary mechanisms through which neuroticism is associated with greater mental distress during major societal stressors.

The corresponding results for the other personality traits are reported in Figures A.5–A.8 S1 Appendix. Overall, these patterns were weaker and less systematic than those observed for neuroticism. While some traits were modestly associated with risk perceptions, protective behaviors, or coping strategies in specific periods, the estimated effects were generally small and often statistically insignificant. Consistent with the limited heterogeneity in mental distress by other personality traits documented above, there was little evidence that these traits operate through strong or persistent behavioral or perceptual associations during the pandemic.

## 4. Discussion

The Big Five personality traits – neuroticism, extraversion, openness, agreeableness, and conscientiousness – are established predictors of mental distress [10,11]. At the onset of the COVID-19 pandemic, a sharp and widespread surge in mental health symptoms was documented worldwide [1,2]. As longitudinal evidence subsequently accumulated, it became clear that this initial surge did not translate into a sustained population-level increase in mental illness [6], since average distress levels partially receded as people adapted to the new circumstances. Yet the degree to which personality traits moderated the trajectory and persistence of mental distress across the full arc of the pandemic — from acute onset through the post-pandemic period — and through which specific mechanisms they operated, remained less well understood, particularly in population-representative U.S. samples. Using longitudinal data from 2019–2024 in the nationally representative Understanding America Study, we examined whether personality traits were associated with the evolution of mental distress during and after this large-scale societal shock, and whether trait-linked differences in risk perception, protective behavior, and coping mapped onto those trajectories.

Our findings point to a highly asymmetric role of personality across the pandemic period. Neuroticism emerged as the primary and most robust correlate of sustained mental distress. Individuals in the highest quartile of neuroticism experienced substantially larger increases in both the level of mental distress, as measured by the continuous PHQ-4 score, and the probability of meeting the threshold for clinical mental distress (PHQ-4 ≥ 6) at the onset of the pandemic. These differences remained statistically significant throughout subsequent phases and into the aftermath of the pandemic. This pattern aligns closely with findings from other longitudinal studies conducted during the pandemic [37]. reported that neuroticism was the strongest personality predictor of pandemic-related distress in a Vermont community sample, and [38] similarly found that higher neuroticism was associated with greater emotional distress during the pandemic in a Finnish cohort. [39] observed an association between neuroticism and mental health symptoms in a Dutch sample, reflecting the trait’s cross-national relevance as a risk factor for mental distress. Using a multi-wave Australian design, [40] extended these findings by documenting that neuroticism predicted not only the acute onset but also the maintenance of distress over the pandemic period – consistent with neuroticism’s known associations with heightened threat appraisal, lower tolerance for uncertainty, and greater susceptibility to anxiety and depression [15,41]. Most pertinent to our own longitudinal findings are [42,43], who both found that neuroticism predicted the persistence of mental health problems well beyond the acute pandemic phase.

The remaining personality traits played a far more limited role. Extraversion, openness, and agreeableness were not systematically associated with differential trajectories of mental distress, and the null results observed in the full sample were not masking substantial heterogeneity across age groups. Conscientiousness exhibited a modest protective association in the continuous PHQ-4 score (particularly among older adults), but these differences were weaker and not robust when a binary distress indicator was used as the outcome variable. This pattern of largely null or weak effects for traits other than neuroticism is consistent with evidence from [40,43], who similarly found that extraversion and agreeableness played minimal roles in predicting longitudinal distress trajectories during the pandemic. These convergent null findings suggest that non-neuroticism traits are unlikely to be meaningful targets for personality-informed intervention in crisis contexts.

Age-stratified analyses further indicate that the vulnerability associated with high neuroticism was broadly distributed across the life course. Young, middle-aged, and older adults with elevated neuroticism all experienced pronounced and persistent increases in symptoms, with only modest differences in magnitude across age groups. This finding contrasts with [12], who report evidence of age moderation in pandemic-related well-being effects. One possible explanation is that a brief screening instrument such as the PHQ-4 may be less sensitive to subtle age-specific differences in symptom profiles or to potential variation in item functioning across age groups. The lack of strong age moderation in our study reinforces the interpretation that neuroticism functions as a relatively stable, life-course-spanning risk factor for mental distress rather than one concentrated at a particular lifetime stage.

The behavioral and perceptual mechanisms documented in our analysis corroborate this interpretation. Individuals high in neuroticism reported higher perceived health and financial risks, greater engagement in protective behaviors, and less adaptive coping strategies – including reduced relaxation and physical activity and increased alcohol consumption. These patterns are consistent with prior evidence that neuroticism is associated with stronger stress responses and shapes both cognitive appraisal and coping behavior under uncertainty [16,24]. Analogous mechanisms were weak or inconsistent for the other personality traits, mirroring the limited heterogeneity observed in mental health outcomes.

An important contextual consideration is that the elevated levels of mental distress observed during the pandemic did not arise in isolation. As [44,45] document using national survey data from the United States and Australia, psychological distress had already been trending upward in the years preceding COVID-19, reflecting a broader secular pattern. From this perspective, pandemic-era distress can be understood as an acceleration of an existing trajectory rather than a wholly new phenomenon. Our findings are consistent with this narrative: mental distress remained above the 2019 baseline nearly four years after the pandemic’s onset, despite the waning of the immediate health threat. This persistence likely reflects both lasting effects of the pandemic and the continuation of pre-existing trends, with personality traits – particularly neuroticism – shaping which individuals were most vulnerable to these compounded stressors.

Our empirical results are subject to several limitations. First, although the PHQ-4 is a validated scale to measure mental distress, it remains self-reported and does not substitute for a clinical diagnosis. It is worth noting, however, that recent contributions suggest that self-report measures may offer important advantages over clinician-administered interviews for capturing subjective psychological distress [46]. Second, despite extensive controls and pre-pandemic baselines, our estimates are correlational and do not establish causal pathways. For example, individuals high in neuroticism may systematically differ in contextual factors – such as living in denser urban areas or holding more precarious employment – which could independently affect risk perceptions and mental distress during a crisis. Establishing causal pathways would require experimental or quasi-experimental designs, which are challenging to implement during a global crisis. Third, our data do not include information on respondents’ use of mental health services during the study period. To the extent that personality traits, particularly neuroticism, are associated with differential uptake of mental health treatment, unobserved treatment use could influence observed PHQ-4 trajectories and potentially attenuate the estimated associations. Fourth, our analyses rely on quartile classifications of personality traits and three broad age groups (18–39, 40–59, 60+). As a robustness check, we re-estimated all main models using quintiles and obtained substantively identical results. Our three age categories reflect meaningful life-course distinctions consistent with prior literature, though different cutoffs may yield modestly different patterns. Fifth, our analysis focused on the United States, and findings may not generalize to settings that differ substantially in their institutional and cultural context. In particular, the absence of a universal health insurance system in the United States may amplify the mental health consequences of health and economic shocks relative to countries where individuals have guaranteed access to medical and psychological care. Readers should therefore exercise caution when extrapolating our findings to contexts with markedly different health care and social protection systems.

Taken together, the evidence suggests that the long-term mental health consequences of the pandemic were not evenly distributed across personality profiles. Rather, elevated and persistent mental distress was concentrated among individuals high in neuroticism, while most other traits were associated with limited or transitory differences. This pattern helps reconcile mixed findings in the broader literature: aggregate averages obscure substantial heterogeneity, with sustained deterioration driven primarily by a psychologically vulnerable subgroup [12,13]. Our findings extend the literature on personality and crisis response, suggesting that neuroticism acts as a psychological amplifier of environmental shocks.

The concentration of persistent mental distress among individuals high in neuroticism has practical implications for intervention, though these must be calibrated carefully to the evidence. A critical point is that elevated neuroticism is a risk factor, not a determinant: most high-neuroticism individuals in our sample did not meet clinical thresholds for mental distress during the pandemic. It would therefore be inappropriate to recommend broad preventive psychological interventions – such as cognitive-behavioral therapy (CBT) – for all individuals scoring high on neuroticism. Such an approach would be inefficient and could pathologize normal variation in personality. Instead, a stepped-care model [47], in which individuals are monitored following a crisis exposure and more intensive interventions are reserved for those who develop clinically significant and persistent distress, would be more appropriate. CBT and emotion regulation training have demonstrated efficacy in reducing neuroticism-linked anxiety and depression [48,49], and could be used within a stepped-care framework.

With respect to early screening, [50,51] caution that universal early psychological debriefing after traumatic events may disrupt natural recovery and, in some cases, increase the risk of PTSD compared to watchful waiting. This evidence suggests restraint in recommending immediate and universal screening or intervention for all crisis-exposed individuals with high neuroticism. Instead, a monitoring-first approach may be preferable, involving structured follow-up assessments and reserving intensive intervention for those who do not recover naturally. Such a strategy retains the potential benefits of personality-informed risk identification while minimizing the risk of unnecessary or counterproductive treatment.

Public health communication during crises presents particular challenges for individuals high in neuroticism, who are more prone to amplifying threat and uncertainty. Rather than simply recommending that messages balance awareness with reassurance, we suggest several practical considerations. Communication should emphasize controllable protective behaviors, such as vaccination, hygiene, and distancing, over uncontrollable risks, consistent with evidence that response-efficacy framing sustains protective behavior while limiting anxiety arousal [52]. Materials could also be pre-tested among high-anxiety samples to avoid unnecessarily distressing language, and tiered information formats may allow individuals to choose the level of detail they receive. Finally, integrating behavioral scientists with expertise in affective risk responses into campaign design from the outset may help anticipate unintended psychological effects.

Beyond individual-level interventions, strengthening population-level resilience is essential for future crises. Pre-crisis strategies include fostering community cohesion and social support networks, which have proven to buffer the mental health impacts of disasters [53], as well as integrating mental health surge capacity into emergency preparedness plans and training front-line workers in basic psychological support and referral pathways. During crises, real-time mental health surveillance and continuity of care, including accessible telehealth services, can support more responsive and targeted action. Together, these structural measures complement individual-level interventions and help address the broader mental health burden generated by large-scale shocks.

In conclusion, neuroticism emerges as the principal personality-linked risk factor for sustained mental distress during the COVID-19 pandemic, a finding that is consistent across time, age groups, and measurement approaches. This highlights the relevance of personality traits for identifying individuals who may require sustained support, while cautioning against universal intervention based solely on personality profiles. Complementing targeted clinical care, broader efforts to strengthen community resilience and mental health infrastructure before and during crises remain important for building a more responsive mental health system.

## Supporting information

S1 Appendix Survey questions, supplementary tables, and supplementary figures. (DOCX)
