# Supplementary material for: How personality traits shape susceptibility to mental distress in times of crisis: Evidence from the COVID-19 pandemic
Source: PLoS One. 2026 Oct 7;21(10):e0358371. doi: 10.1371/journal.pone.0358371 (PMC13645027; doi:10.1371/journal.pone.0358371)
Supplement: Supplementary file 1 [file pone.0358371.s001.docx]

**APPENDIX**

**PHQ-4 Item Inventory and Scoring**

**Questions**: Over the past fourteen days, how often have you been bothered by any of the following problems?

a) Feeling nervous, anxious, or on edge

b) Not being able to stop or control worrying

c) Feeling down, depressed, or hopeless

d) Little interest or pleasure in doing things

**Answer scale**:

0**=**Not at all

1**=**Several days

2**=**More than half the days

3**=**Nearly every day

**Scoring**:

The total PHQ-4 score is obtained by summing scores across itmes a)-d).

**Risk Perception Questions**

The risk perception questions reported below were asked in all the waves of the Understanding Coronavirus in America Survey (UCAS), from wave 1 (March 2020) to wave 34 (May-July 2023).

Respondents answered these questions by entering a number between 0% and 100%.

1) On a scale of 0 to 100 percent, what is the chance that you will get the coronavirus in the

next three months? If you’re not sure, please give your best guess.

2) If you do get the coronavirus, what is the percent chance you will be hospitalized (spend

at least one night in the hospital) from it? If you’re not sure, please give your best guess.

3) If you do get the coronavirus, what is the percent chance you will die from it? If you’re

not sure, please give your best guess.

4) The coronavirus may cause economic challenges for some people regardless of whether they are actually infected.

What is the percent chance that you will lose your job because of the coronavirus

within the next three months?

5) The coronavirus may cause economic challenges for some people regardless of whether they are actually infected.

What is the percent chance you will run out of money because of the coronavirus

in the next three months?

**Protective Behavior Questions**

The protective behavior items reported below were asked in waves 2 (April 2020) to 30 (September–October 2021) of the Understanding Coronavirus in America Survey (UCAS).

For each item, answer options were:

1- Yes

2- No

3- Unsure

Which of the following have you done in the last seven days to keep yourself safe from

coronavirus?

- Washed your hands with soap or used hand sanitizer several times per day
- Avoided contact with people who could be high-risk
- Avoided public spaces, gatherings, or crowds
- Worn a mask or other face covering

**Potential Coping Behavior Questions**

The potential coping behavior items reported below were asked in waves 2 (April 2020) to 30 (September–October 2021) of the Understanding Coronavirus in America Survey (UCAS).

Respondents answered these questions by entering a number between 0 and 7.

Out of the past 7 days, what is your best estimate of the number of days that you did

each of the following activities?

- Drank alcohol
- Used cannabis products such as marijuana
- Used recreational drugs other than alcohol or cannabis products
- Meditated
- Got extra exercise
- Made time to relax

**Big-5 Item Inventory and Scoring**

**Question**:

“This first section consists of a series of statements that may or may not apply to you. For each statement, rate how much you agree or disagree that the statement accurately describes you. There are no right or wrong answers. I am someone who…”

**Answer scale**:

1**=**Disagree strongly

2**=**Disagree a little

3**=**Neither agree nor disagree

4**=**Agree a little

5**=**Agree strongly

**Scoring**:

Key items that are bold, italicized, and underlined (e.g., ***E***) should be reverse-scored. To reverse score an item, change 1 to 5, 2 to 4, 4 to 2, and 5 to 1. After reverse scoring, find the total score for N (Neuroticism), E (Extraversion), O (Openness), A (Agreeableness), and C (Conscientiousness) by summing items’ scores.

1	Is talkative [E]

2	Tends to find fault with others [***A***]

3	Does a thorough job [C]

4	Is depressed, blue [N]

5	Is original, comes up with new ideas [O]

6	Is reserved [***E***]

7	Is helpful and unselfish with others [A]

8	Can be somewhat careless [***C***]

9	Is relaxed, handles stress well [***N***]

10	Is curious about many different things [O]

11	Is full of energy [E]

12	Starts quarrels with others [***A***]

13	Is a reliable worker [C]

14	Can be tense [N]

15	Is ingenious, a deep thinker [O]

16	Generates a lot of enthusiasm [E]

17	Has a forgiving nature [A]

18	Tends to be disorganized [***C***]

19	Worries a lot [N]

20	Has an active imagination [O]

21	Tends to be quiet [***E***]

22	Is generally trusting [A]

23	Tends to be lazy [***C***]

24	Is emotionally stable, not easily upset [***N***]

25	Is inventive [O]

26	Has an assertive personality [E]

27	Can be cold and aloof [***A***]

28	Perseveres until the task is finished [C]

29	Can be moody [N]

30	Values artistic, aesthetic experiences [O]

31	Is sometimes shy, inhibited [***E***]

32	Is considerate and kind to almost everyone [A]

33	Does things efficiently [C]

34	Remains calm in tense situations [***N***]

35	Prefers work that is routine [***O***]

36	Is outgoing, sociable [E]

37	Is sometimes rude to others [***A***]

38	Makes plans and follows through with them [C]

39	Gets nervous easily [N]

40	Likes to reflect, play with ideas [O]

41	Has few artistic interests [***O***]

42	Likes to cooperate with others [A]

43	Is easily distracted [***C***]

44	Is sophisticated in art, music, or literature [O]

The inventory and scoring system are based on:

McCrae, R. R. (2004). Human nature and culture: A trait perspective. *Journal of Research in Personality, 38,* 3-14.

McCrae, R. R., & Costa. P. T. Jr. (1999). A five-factor theory of personality. In L. A. Pervin, & O. P. John (Eds.), *Handbook of personality: Theory and research.* New York: Guilford Press.

John, O. P. & Srivastava, S. (1999). The Big Five trait taxonomy: History, measurement, and theoretical perspectives. In L. A. Pervin, & O. P. John (Eds.), *Handbook of personality: Theory and research.* New York: Guilford Press.

**Table A.1 – Baseline Individual Characteristics**

| **Variable** | **Proportion or Mean (sd)** | **Range** |
| --- | --- | --- |
|  |  |  |
| Female | 59.01 | 0-1 |
| Male | 40.99 | 0-1 |
|  |  |  |
| White | 59.08 | 0-1 |
| Black | 8.39 | 0-1 |
| Other | 11.96 | 0-1 |
| Hispanic | 20.57 | 0-1 |
|  |  |  |
| Age 18-39 | 36.45 | 0-1 |
| Age 40-49 | 17.79 | 0-1 |
| Age 50-59 | 17.82 | 0-1 |
| Age 60+ | 27.94 | 0-1 |
|  |  |  |
| High school or less | 22.63 | 0-1 |
| Some college | 35.58 | 0-1 |
| College degree or more | 41.79 | 0-1 |
|  |  |  |
| Married | 52.01 | 0-1 |
| Sep/Div/Wid | 19.58 | 0-1 |
| Never married | 28.41 | 0-1 |
|  |  |  |
| Household income <$30,000 | 26.13 | 0-1 |
| Household income $30,000-$59,999 | 25.61 | 0-1 |
| Household income $60,000-$99,999 | 23.97 | 0-1 |
| Household income $100,000+ | 24.29 | 0-1 |
|  |  |  |
| Neuroticism | 22.23 (3.81) | 8-40 |
| Extraversion | 25.46 (3.67) | 8-40 |
| Openness | 37.08 (5.88) | 10-50 |
| Agreeableness | 35.54 (6.04) | 9-45 |
| Conscientiousness | 34.92 (5.33) | 9-45 |
|  |  |  |
| PHQ-4 score | 1.55 (1.96) | 0-12 |

Individual demographics and traits observed at baseline (pre-pandemic period); N=4,030.

**Table A.2 – Standardized Differences by Level of Panel Participation**

|  | **Standardized Differences** | |
| --- | --- | --- |
| **Variable** | **Low vs. Very High** | **Medium/High vs. Very High** |
|  |  |  |
| Female | 0.06 | 0.05 |
| Male | -0.06 | -0.05 |
|  |  |  |
| White | -0.33 | -0.24 |
| Black | 0.10 | 0.07 |
| Other | -0.03 | 0.00 |
| Hispanic | 0.35 | 0.24 |
|  |  |  |
| Age 18-39 | 0.57 | 0.36 |
| Age 40-49 | -0.03 | 0.14 |
| Age 50-59 | -0.17 | -0.14 |
| Age 60+ | -0.44 | -0.38 |
|  |  |  |
| High school or less | 0.19 | 0.10 |
| Some college | 0.09 | 0.08 |
| College degree or more | -0.25 | -0.16 |
|  |  |  |
| Married | -0.19 | -0.15 |
| Sep/Div/Wid | -0.14 | -0.07 |
| Never married | 0.33 | 0.23 |
|  |  |  |
| Household income <$30,000 | 0.16 | 0.16 |
| Household income $30,000-$59,999 | 0.04 | 0.00 |
| Household income $60,000-$99,999 | -0.08 | -0.05 |
| Household income $100,000+ | -0.13 | -0.12 |
|  |  |  |
| Neuroticism | 0.17 | 0.12 |
| Extraversion | 0.15 | 0.04 |
| Openness | 0.15 | 0.10 |
| Agreeableness | -0.02 | 0.01 |
| Conscientiousness | -0.24 | -0.20 |
|  |  |  |
| PHQ-4 score | 0.17 | 0.16 |

Standardized differences are defined as differences in group means scaled by pooled standard deviations. All reported differences are relative to the group with very high participation (those who completed 34-36 waves). N=4,030.

**Table A.3 - Multinomial Logit for Panel Participation Categories**

|  | Low: | Medium/High: | Very High |
| --- | --- | --- | --- |
|  | 2-22 | 23-33 | 34-36 |
| VARIABLES | waves | waves | waves |
|  |  |  |  |
| Male | 0.015 | 0.006 | -0.021 |
|  | (0.015) | (0.015) | (0.016) |
| Black | 0.070** | 0.016 | -0.086*** |
|  | (0.027) | (0.027) | (0.029) |
| Other | 0.002 | 0.003 | -0.005 |
|  | (0.021) | (0.022) | (0.024) |
| Hispanic | 0.064*** | 0.024 | -0.089*** |
|  | (0.019) | (0.019) | (0.021) |
| Age 40-49 | -0.118*** | 0.031 | 0.087*** |
|  | (0.021) | (0.023) | (0.023) |
| Age 50-59 | -0.134*** | -0.065*** | 0.199*** |
|  | (0.022) | (0.022) | (0.024) |
| Age 60+ | -0.165*** | -0.100*** | 0.265*** |
|  | (0.020) | (0.020) | (0.022) |
| Some college | -0.036* | 0.010 | 0.027 |
|  | (0.019) | (0.019) | (0.021) |
| College degree or more | -0.085*** | -0.012 | 0.097*** |
|  | (0.020) | (0.020) | (0.022) |
| Separated/Divorced/Widowed | -0.013 | 0.008 | 0.005 |
|  | (0.019) | (0.020) | (0.021) |
| Never married | -0.001 | -0.005 | 0.006 |
|  | (0.018) | (0.018) | (0.021) |
| Household income $30,000-$59,999 | 0.012 | -0.023 | 0.010 |
|  | (0.019) | (0.020) | (0.022) |
| Household income $60,000-$99,999 | 0.002 | -0.022 | 0.020 |
|  | (0.020) | (0.022) | (0.023) |
| Household income $100,000+ | 0.010 | -0.036 | 0.025 |
|  | (0.022) | (0.023) | (0.025) |
| Neuroticism | 0.014 | -0.003 | -0.010 |
|  | (0.009) | (0.009) | (0.010) |
| Extraversion | 0.026*** | -0.003 | -0.024*** |
|  | (0.007) | (0.007) | (0.008) |
| Openness | 0.023*** | 0.012 | -0.035*** |
|  | (0.008) | (0.008) | (0.008) |
| Agreeableness | 0.010 | 0.015* | -0.025*** |
|  | (0.008) | (0.008) | (0.009) |
| Conscientiousness | -0.025*** | -0.021*** | 0.046*** |
|  | (0.008) | (0.008) | (0.009) |
| PHQ-4 Score | 0.001 | 0.005 | -0.007 |
|  | (0.004) | (0.004) | (0.004) |
|  |  |  |  |
| Observations | 4,030 | 4,030 | 4,030 |

Robust standard errors in parentheses; *** p<0.01, ** p<0.05, * p<0.1

**Table A.4 – Linear Regressions for PHQ-4 Score: Full Set of Estimated Coefficients**

**with Neuroticism Quartile Indicators Interacted with Period Indicators**

| Mar20/Jun20 | 0.159^**^ |
| --- | --- |
|  | (0.077) |
| Jul20/Oct20 | -0.068 |
|  | (0.078) |
| Nov20/Feb21 | -0.033 |
|  | (0.080) |
| Mar21/Jan22 | -0.169^**^ |
|  | (0.077) |
| Feb22/Apr23 | -0.131 |
|  | (0.080) |
| May23/Feb24 | -0.114 |
|  | (0.086) |
| Neuroticism Q2 | 0.223^**^ |
|  | (0.098) |
| Neuroticism_Q3 | 0.684^***^ |
|  | (0.134) |
| Neuroticism Q4 | 1.588^***^ |
|  | (0.172) |
| Mar20/Jun20 × Neuroticism Q2 | 0.420^***^ |
|  | (0.109) |
| Mar20/Jun20 × Neuroticism Q3 | 0.714^***^ |
|  | (0.145) |
| Mar20/Jun20 × Neuroticism Q4 | 1.113^***^ |
|  | (0.177) |
| Jul20/Oct20 × Neuroticism Q2 | 0.350^***^ |
|  | (0.109) |
| Jul20/Oct20 × Neuroticism Q3 | 0.653^***^ |
|  | (0.146) |
| Jul20/Oct20 × Neuroticism Q4 | 1.053^***^ |
|  | (0.184) |
| Nov20/Feb21 × Neuroticism Q2 | 0.363^***^ |
|  | (0.113) |
| Nov20/Feb21× Neuroticism Q3 | 0.572^***^ |
|  | (0.152) |
| Nov20/Feb21 × Neuroticism Q4 | 1.041^***^ |
|  | (0.190) |
| Mar21/Jan22 × Neuroticism Q2 | 0.333^***^ |
|  | (0.110) |
| Mar21/Jan22 × Neuroticism Q3 | 0.447^***^ |
|  | (0.147) |
| Mar21/Jan22 × Neuroticism Q4 | 0.911^***^ |
|  | (0.186) |
| Feb22/Apr23 × Neuroticism Q2 | 0.245^**^ |
|  | (0.111) |
| Feb22/Apr23 × Neuroticism Q3 | 0.331^**^ |
|  | (0.150) |
| Feb22/Apr23 × Neuroticism Q4 | 0.721^***^ |
|  | (0.188) |
| May23/Feb24 × Neuroticism Q2 | 0.173 |
|  | (0.123) |
| May23/Feb24 × Neuroticism Q3 | 0.334^**^ |
|  | (0.164) |
| May23/Feb24 × Neuroticism Q4 | 0.845^***^ |
|  | (0.208) |
| Extraversion Q2 | -0.166^*^ |
|  | (0.101) |
| Extraversion Q3 | -0.240^**^ |
|  | (0.096) |
| Extraversion Q4 | -0.190^*^ |
|  | (0.098) |
| Openness Q2 | 0.116 |
|  | (0.098) |
| Openness Q3 | 0.255^***^ |
|  | (0.098) |
| Openness Q4 | 0.492^***^ |
|  | (0.104) |
| Agreeableness Q2 | 0.125 |
|  | (0.102) |
| Agreeableness Q3 | 0.226^**^ |
|  | (0.104) |
| Agreeableness Q4 | 0.317^***^ |
|  | (0.111) |
| Conscientiousness Q2 | -0.280^***^ |
|  | (0.107) |
| Conscientiousness Q3 | -0.340^***^ |
|  | (0.107) |
| Conscientiousness Q4 | -0.435^***^ |
|  | (0.110) |
| Male | -0.202^***^ |
|  | (0.072) |
| Black | -0.497^***^ |
|  | (0.126) |
| Other | -0.012 |
|  | (0.122) |
| Hispanic | 0.017 |
|  | (0.100) |
| Age 40-49 | -0.267^**^ |
|  | (0.106) |
| Age 50-59 | -0.333^***^ |
|  | (0.112) |
| Age 60+ | -0.681^***^ |
|  | (0.099) |
| Some college | 0.219^**^ |
|  | (0.106) |
| College degree or more | 0.342^***^ |
|  | (0.105) |
| Separated/Divorced/Widowed | 0.341^***^ |
|  | (0.095) |
| Never married | 0.506^***^ |
|  | (0.100) |
| Household income $30,000-$59,999 | -0.314^***^ |
|  | (0.105) |
| Household income $60,000-$99,999 | -0.538^***^ |
|  | (0.109) |
| Household income $100,000+ | -0.760^***^ |
|  | (0.110) |
| Constant | 1.354^***^ |
|  | (0.207) |
| *N* | 106,668 |

Clustered standard errors in parentheses; *** p<0.01, ** p<0.05, * p<0.1

**Table A.5 – Linear Regressions for PHQ-4 Score: Full Set of Estimated Coefficients**

**with Extraversion Quartile Indicators Interacted with Period Indicators**

| Mar20/Jun20 | 0.710^***^ |
| --- | --- |
|  | (0.097) |
| Jul20/Oct20 | 0.447^***^ |
|  | (0.100) |
| Nov20/Feb21 | 0.477^***^ |
|  | (0.104) |
| Mar21/Jan22 | 0.296^***^ |
|  | (0.103) |
| Feb22/Apr23 | 0.229^**^ |
|  | (0.103) |
| May23/Feb24 | 0.235^**^ |
|  | (0.116) |
| Extraversion Q2 | -0.193 |
|  | (0.152) |
| Extraversion Q3 | -0.156 |
|  | (0.132) |
| Extraversion Q4 | -0.145 |
|  | (0.143) |
| Mar20/Jun20 × Extraversion Q2 | -0.020 |
|  | (0.166) |
| Mar20/Jun20 × Extraversion Q3 | 0.029 |
|  | (0.144) |
| Mar20/Jun20 × Extraversion Q4 | -0.024 |
|  | (0.154) |
| Jul20/Oct20 × Extraversion Q2 | 0.051 |
|  | (0.171) |
| Jul20/Oct20 × Extraversion Q3 | -0.084 |
|  | (0.147) |
| Jul20/Oct20 × Extraversion Q4 | -0.042 |
|  | (0.158) |
| Nov20/Feb21 × Extraversion Q2 | 0.035 |
|  | (0.177) |
| Nov20/Feb21× Extraversion Q3 | -0.137 |
|  | (0.153) |
| Nov20/Feb21 × Extraversion Q4 | -0.029 |
|  | (0.162) |
| Mar21/Jan22 × Extraversion Q2 | 0.058 |
|  | (0.175) |
| Mar21/Jan22 × Extraversion Q3 | -0.186 |
|  | (0.148) |
| Mar21/Jan22 × Extraversion Q4 | -0.097 |
|  | (0.157) |
| Feb22/Apr23 × Extraversion Q2 | 0.019 |
|  | (0.175) |
| Feb22/Apr23 × Extraversion Q3 | -0.122 |
|  | (0.151) |
| Feb22/Apr23 × Extraversion Q4 | -0.076 |
|  | (0.158) |
| May23/Feb24 × Extraversion Q2 | 0.003 |
|  | (0.190) |
| May23/Feb24 × Extraversion Q3 | -0.004 |
|  | (0.171) |
| May23/Feb24 × Extraversion Q4 | -0.090 |
|  | (0.175) |
| Neuroticism Q2 | 0.563^***^ |
|  | (0.074) |
| Neuroticism Q3 | 1.243^***^ |
|  | (0.095) |
| Neuroticism Q4 | 2.558^***^ |
|  | (0.121) |
| Openness Q2 | 0.117 |
|  | (0.098) |
| Openness Q3 | 0.256^***^ |
|  | (0.098) |
| Openness Q4 | 0.492^***^ |
|  | (0.104) |
| Agreeableness Q2 | 0.125 |
|  | (0.102) |
| Agreeableness Q3 | 0.226^**^ |
|  | (0.104) |
| Agreeableness Q4 | 0.317^***^ |
|  | (0.111) |
| Conscientiousness Q2 | -0.280^***^ |
|  | (0.107) |
| Conscientiousness Q3 | -0.340^***^ |
|  | (0.107) |
| Conscientiousness Q4 | -0.435^***^ |
|  | (0.110) |
| Male | -0.201^***^ |
|  | (0.072) |
| Black | -0.497^***^ |
|  | (0.126) |
| Other | -0.011 |
|  | (0.123) |
| Hispanic | 0.017 |
|  | (0.100) |
| Age 40-49 | -0.266^**^ |
|  | (0.106) |
| Age 50-59 | -0.332^***^ |
|  | (0.112) |
| Age 60+ | -0.681^***^ |
|  | (0.099) |
| Some college | 0.220^**^ |
|  | (0.106) |
| College degree or more | 0.342^***^ |
|  | (0.105) |
| Separated/Divorced/Widowed | 0.342^***^ |
|  | (0.095) |
| Never married | 0.507^***^ |
|  | (0.100) |
| Household income $30,000-$59,999 | -0.314^***^ |
|  | (0.105) |
| Household income $60,000-$99,999 | -0.539^***^ |
|  | (0.109) |
| Household income $100,000+ | -0.760^***^ |
|  | (0.111) |
| Constant | 0.875^***^ |
|  | (0.209) |
| *N* | 106,668 |

Clustered standard errors in parentheses; *** p<0.01, ** p<0.05, * p<0.1

**Table A.6 – Linear Regressions for PHQ-4 Score: Full Set of Estimated Coefficients**

**with Openness Quartile Indicators Interacted with Period Indicators**

| Mar20/Jun20 | 0.662^***^ |
| --- | --- |
|  | (0.106) |
| Jul20/Oct20 | 0.387^***^ |
|  | (0.109) |
| Nov20/Feb21 | 0.503^***^ |
|  | (0.114) |
| Mar21/Jan22 | 0.328^***^ |
|  | (0.112) |
| Feb22/Apr23 | 0.311^***^ |
|  | (0.112) |
| May23/Feb24 | 0.389^***^ |
|  | (0.126) |
| Openness Q2 | 0.211 |
|  | (0.129) |
| Openness Q3 | 0.291^*^ |
|  | (0.152) |
| Openness Q4 | 0.447^***^ |
|  | (0.157) |
| Mar20/Jun20 × Openness Q2 | -0.008 |
|  | (0.146) |
| Mar20/Jun20 × Openness Q3 | 0.069 |
|  | (0.165) |
| Mar20/Jun20 × Openness Q4 | 0.115 |
|  | (0.166) |
| Jul20/Oct20 × Openness Q2 | -0.015 |
|  | (0.149) |
| Jul20/Oct20 × Openness Q3 | 0.036 |
|  | (0.169) |
| Jul20/Oct20 × Openness Q4 | 0.151 |
|  | (0.171) |
| Nov20/Feb21 × Openness Q2 | -0.176 |
|  | (0.154) |
| Nov20/Feb21× Openness Q3 | -0.145 |
|  | (0.173) |
| Nov20/Feb21 × Openness Q4 | 0.078 |
|  | (0.177) |
| Mar21/Jan22 × Openness Q2 | -0.136 |
|  | (0.151) |
| Mar21/Jan22 × Openness Q3 | -0.120 |
|  | (0.170) |
| Mar21/Jan22 × Openness Q4 | -0.090 |
|  | (0.173) |
| Feb22/Apr23 × Openness Q2 | -0.231 |
|  | (0.150) |
| Feb22/Apr23 × Openness Q3 | -0.077 |
|  | (0.172) |
| Feb22/Apr23 × Openness Q4 | -0.206 |
|  | (0.174) |
| May23/Feb24 × Openness Q2 | -0.360^**^ |
|  | (0.170) |
| May23/Feb24 × Openness Q3 | -0.106 |
|  | (0.188) |
| May23/Feb24 × Openness Q4 | -0.266 |
|  | (0.191) |
| Neuroticism Q2 | 0.563^***^ |
|  | (0.074) |
| Neuroticism Q3 | 1.243^***^ |
|  | (0.095) |
| Neuroticism Q4 | 2.559^***^ |
|  | (0.121) |
| Extraversion Q2 | -0.167^*^ |
|  | (0.101) |
| Extraversion Q3 | -0.240^**^ |
|  | (0.096) |
| Extraversion Q4 | -0.190^*^ |
|  | (0.098) |
| Agreeableness Q2 | 0.125 |
|  | (0.102) |
| Agreeableness Q3 | 0.225^**^ |
|  | (0.104) |
| Agreeableness Q4 | 0.316^***^ |
|  | (0.111) |
| Conscientiousness Q2 | -0.279^***^ |
|  | (0.107) |
| Conscientiousness Q3 | -0.339^***^ |
|  | (0.107) |
| Conscientiousness Q4 | -0.435^***^ |
|  | (0.110) |
| Male | -0.201^***^ |
|  | (0.072) |
| Black | -0.497^***^ |
|  | (0.126) |
| Other | -0.011 |
|  | (0.122) |
| Hispanic | 0.018 |
|  | (0.100) |
| Age 40-49 | -0.266^**^ |
|  | (0.106) |
| Age 50-59 | -0.333^***^ |
|  | (0.112) |
| Age 60+ | -0.681^***^ |
|  | (0.099) |
| Some college | 0.220^**^ |
|  | (0.106) |
| College degree or more | 0.342^***^ |
|  | (0.105) |
| Separated/Divorced/Widowed | 0.341^***^ |
|  | (0.095) |
| Never married | 0.505^***^ |
|  | (0.100) |
| Household income $30,000-$59,999 | -0.314^***^ |
|  | (0.105) |
| Household income $60,000-$99,999 | -0.539^***^ |
|  | (0.109) |
| Household income $100,000+ | -0.761^***^ |
|  | (0.111) |
| Constant | 0.879^***^ |
|  | (0.209) |
| *N* | 106,668 |

Clustered standard errors in parentheses; *** p<0.01, ** p<0.05, * p<0.1

**Table A.7 – Linear Regressions for PHQ-4 Score: Full Set of Estimated Coefficients**

**with Agreeableness Quartile Indicators Interacted with Period Indicators**

| Mar20/Jun20 | 0.691^***^ |
| --- | --- |
|  | (0.115) |
| Jul20/Oct20 | 0.477^***^ |
|  | (0.118) |
| Nov20/Feb21 | 0.508^***^ |
|  | (0.124) |
| Mar21/Jan22 | 0.307^**^ |
|  | (0.121) |
| Feb22/Apr23 | 0.237^*^ |
|  | (0.124) |
| May23/Feb24 | 0.367^***^ |
|  | (0.138) |
| Openness Q2 | 0.045 |
|  | (0.146) |
| Openness Q3 | 0.299^**^ |
|  | (0.135) |
| Openness Q4 | 0.497^***^ |
|  | (0.160) |
| Mar20/Jun20 × Agreeableness Q2 | 0.189 |
|  | (0.161) |
| Mar20/Jun20 × Agreeableness Q3 | -0.016 |
|  | (0.147) |
| Mar20/Jun20 × Agreeableness Q4 | -0.130 |
|  | (0.168) |
| Jul20/Oct20 × Agreeableness Q2 | 0.052 |
|  | (0.165) |
| Jul20/Oct20 × Agreeableness Q3 | -0.077 |
|  | (0.151) |
| Jul20/Oct20 × Agreeableness Q4 | -0.180 |
|  | (0.172) |
| Nov20/Feb21 × Agreeableness Q2 | 0.051 |
|  | (0.171) |
| Nov20/Feb21× Agreeableness Q3 | -0.099 |
|  | (0.158) |
| Nov20/Feb21 × Agreeableness Q4 | -0.212 |
|  | (0.177) |
| Mar21/Jan22 × Agreeableness Q2 | 0.052 |
|  | (0.167) |
| Mar21/Jan22 × Agreeableness Q3 | -0.105 |
|  | (0.154) |
| Mar21/Jan22 × Agreeableness Q4 | -0.209 |
|  | (0.173) |
| Feb22/Apr23 × Agreeableness Q2 | 0.086 |
|  | (0.171) |
| Feb22/Apr23 × Agreeableness Q3 | -0.081 |
|  | (0.157) |
| Feb22/Apr23 × Agreeableness Q4 | -0.217 |
|  | (0.174) |
| May23/Feb24 × Agreeableness Q2 | -0.089 |
|  | (0.190) |
| May23/Feb24 × Agreeableness Q3 | -0.232 |
|  | (0.174) |
| May23/Feb24 × Agreeableness Q4 | -0.321^*^ |
|  | (0.190) |
| Neuroticism Q2 | 0.563^***^ |
|  | (0.074) |
| Neuroticism Q3 | 1.243^***^ |
|  | (0.095) |
| Neuroticism Q4 | 2.559^***^ |
|  | (0.121) |
| Extraversion Q2 | -0.166^*^ |
|  | (0.101) |
| Extraversion Q3 | -0.240^**^ |
|  | (0.096) |
| Extraversion Q4 | -0.190^*^ |
|  | (0.098) |
| Openness Q2 | 0.117 |
|  | (0.098) |
| Openness Q3 | 0.256^***^ |
|  | (0.098) |
| Openness Q4 | 0.492^***^ |
|  | (0.104) |
| Conscientiousness Q2 | -0.280^***^ |
|  | (0.107) |
| Conscientiousness Q3 | -0.340^***^ |
|  | (0.107) |
| Conscientiousness Q4 | -0.435^***^ |
|  | (0.110) |
| Male | -0.201^***^ |
|  | (0.072) |
| Black | -0.496^***^ |
|  | (0.126) |
| Other | -0.011 |
|  | (0.123) |
| Hispanic | 0.017 |
|  | (0.100) |
| Age 40-49 | -0.267^**^ |
|  | (0.106) |
| Age 50-59 | -0.333^***^ |
|  | (0.112) |
| Age 60+ | -0.681^***^ |
|  | (0.099) |
| Some college | 0.220^**^ |
|  | (0.106) |
| College degree or more | 0.341^***^ |
|  | (0.105) |
| Separated/Divorced/Widowed | 0.342^***^ |
|  | (0.095) |
| Never married | 0.507^***^ |
|  | (0.100) |
| Household income $30,000-$59,999 | -0.314^***^ |
|  | (0.105) |
| Household income $60,000-$99,999 | -0.539^***^ |
|  | (0.109) |
| Household income $100,000+ | -0.759^***^ |
|  | (0.111) |
| Constant | 0.859^***^ |
|  | (0.208) |
| *N* | 106,668 |

Clustered standard errors in parentheses; *** p<0.01, ** p<0.05, * p<0.1

**Table A.8 – Linear Regressions for PHQ-4 Score: Full Set of Estimated Coefficients**

**with Conscientiousness Quartile Indicators Interacted with Period Indicators**

| Mar20/Jun20 | 0.928^***^ |
| --- | --- |
|  | (0.121) |
| Jul20/Oct20 | 0.699^***^ |
|  | (0.126) |
| Nov20/Feb21 | 0.687^***^ |
|  | (0.131) |
| Mar21/Jan22 | 0.551^***^ |
|  | (0.129) |
| Feb22/Apr23 | 0.368^***^ |
|  | (0.131) |
| May23/Feb24 | 0.394^***^ |
|  | (0.144) |
| Openness Q2 | -0.024 |
|  | (0.164) |
| Openness Q3 | -0.100 |
|  | (0.151) |
| Openness Q4 | 0.062 |
|  | (0.151) |
| Mar20/Jun20 × Conscientiousness Q2 | -0.215 |
|  | (0.179) |
| Mar20/Jun20 × Conscientiousness Q3 | -0.203 |
|  | (0.162) |
| Mar20/Jun20 × Conscientiousness Q4 | -0.515^***^ |
|  | (0.157) |
| Jul20/Oct20 × Conscientiousness Q2 | -0.272 |
|  | (0.183) |
| Jul20/Oct20 × Conscientiousness Q3 | -0.282^*^ |
|  | (0.169) |
| Jul20/Oct20 × Conscientiousness Q4 | -0.569^***^ |
|  | (0.160) |
| Nov20/Feb21 × Conscientiousness Q2 | -0.285 |
|  | (0.189) |
| Nov20/Feb21× Conscientiousness Q3 | -0.243 |
|  | (0.174) |
| Nov20/Feb21 × Conscientiousness Q4 | -0.474^***^ |
|  | (0.167) |
| Mar21/Jan22 × Conscientiousness Q2 | -0.378^**^ |
|  | (0.187) |
| Mar21/Jan22 × Conscientiousness Q3 | -0.317^*^ |
|  | (0.170) |
| Mar21/Jan22 × Conscientiousness Q4 | -0.575^***^ |
|  | (0.162) |
| Feb22/Apr23 × Conscientiousness Q2 | -0.175 |
|  | (0.188) |
| Feb22/Apr23 × Conscientiousness Q3 | -0.189 |
|  | (0.172) |
| Feb22/Apr23 × Conscientiousness Q4 | -0.390^**^ |
|  | (0.166) |
| May23/Feb24 × Conscientiousness Q2 | -0.140 |
|  | (0.205) |
| May23/Feb24 × Conscientiousness Q3 | -0.189 |
|  | (0.191) |
| May23/Feb24 × Conscientiousness Q4 | -0.427^**^ |
|  | (0.179) |
| Neuroticism Q2 | 0.563^***^ |
|  | (0.074) |
| Neuroticism Q3 | 1.244^***^ |
|  | (0.095) |
| Neuroticism Q4 | 2.559^***^ |
|  | (0.121) |
| Extraversion Q2 | -0.166^*^ |
|  | (0.101) |
| Extraversion Q3 | -0.240^**^ |
|  | (0.096) |
| Extraversion Q4 | -0.190^*^ |
|  | (0.098) |
| Openness Q2 | 0.117 |
|  | (0.098) |
| Openness Q3 | 0.256^***^ |
|  | (0.098) |
| Openness Q4 | 0.492^***^ |
|  | (0.104) |
| Agreeableness Q2 | 0.125 |
|  | (0.102) |
| Agreeableness Q3 | 0.226^**^ |
|  | (0.104) |
| Agreeableness Q4 | 0.317^***^ |
|  | (0.111) |
| Male | -0.201^***^ |
|  | (0.072) |
| Black | -0.497^***^ |
|  | (0.126) |
| Other | -0.012 |
|  | (0.123) |
| Hispanic | 0.017 |
|  | (0.100) |
| Age 40-49 | -0.267^**^ |
|  | (0.106) |
| Age 50-59 | -0.333^***^ |
|  | (0.112) |
| Age 60+ | -0.681^***^ |
|  | (0.099) |
| Some college | 0.220^**^ |
|  | (0.106) |
| College degree or more | 0.342^***^ |
|  | (0.105) |
| Separated/Divorced/Widowed | 0.342^***^ |
|  | (0.095) |
| Never married | 0.507^***^ |
|  | (0.100) |
| Household income $30,000-$59,999 | -0.314^***^ |
|  | (0.105) |
| Household income $60,000-$99,999 | -0.538^***^ |
|  | (0.109) |
| Household income $100,000+ | -0.759^***^ |
|  | (0.111) |
| Constant | 0.660^***^ |
|  | (0.213) |
| *N* | 106,668 |

Clustered standard errors in parentheses; *** p<0.01, ** p<0.05, * p<0.1

**Figure A.1: Predicted PHQ-4 Score (Pred[PHQ-4]) and Predicted Probability of Mental Distress (Pred[PHQ-4≥6]) over Time by Extraversion and Age**

Note: The figures in the left column refer to the PHQ-4 score; the reported results are obtained from linear regressions, as in Figure 1, estimated separately for each age group. The figures in the right column refer to mental distress (PHQ-4 score ≥ 6); the reported results are obtained from logistic regressions, as in Figure 2, estimated separately for each age group. Relative to the linear and logistic regressions in Figures 1 and 2, Extraversion quartile indicators are replaced with a binary indicator for being above the median level of Extraversion in order to enhance statistical power.

**Figure A.2: Predicted PHQ4 Score (Pred[PHQ-4]) and Predicted Probability of Mental Distress (Pred[PHQ-4≥6]) over Time by Openness and Age**

Note: The figures in the left column refer to the PHQ-4 score; the reported results are obtained from linear regressions, as in Figure 1, estimated separately for each age group. The figures in the right column refer to mental distress (PHQ-4 score ≥ 6); the reported results are obtained from logistic regressions, as in Figure 2, estimated separately for each age group. Relative to the linear and logistic regressions in Figures 1 and 2, openness quartile indicators are replaced with a binary indicator for being above the median level of openness in order to enhance statistical power.

**Figure A.3: Predicted PHQ-4 Score (Pred[PHQ-4]) and Predicted Probability of Mental Distress (Pred[PHQ-4≥6]) over Time by Agreeableness and Age**

Note: The figures in the left column refer to the PHQ-4 score; the reported results are obtained from linear regressions, as in Figure 1, estimated separately for each age group. The figures in the right column refer to mental distress (PHQ-4 score ≥ 6); the reported results are obtained from logistic regressions, as in Figure 2, estimated separately for each age group. Relative to the linear and logistic regressions in Figures 1 and 2, agreeableness quartile indicators are replaced with a binary indicator for being above the median level of agreeableness in order to enhance statistical power.

**Figure A.4: Predicted PHQ-4 Score (Pred[PHQ-4]) and Predicted Probability of Mental Distress (Pred[PHQ-4≥6]) over Time by Conscientiousness and Age**

Note: The figures in the left column refer to the PHQ-4 score; the reported results are obtained from linear regressions, as in Figure 1, estimated separately for each age group. The figures in the right column refer to mental distress (PHQ-4 score ≥ 6); the reported results are obtained from logistic regressions, as in Figure 2, estimated separately for each age group. Relative to the linear and logistic regressions in Figures 1 and 2, conscientiousness quartile indicators are replaced with a binary indicator for being above the median level of conscientiousness in order to enhance statistical power.

**Figure A.5: Marginal Effects of Extraversion on Risk Perceptions, Adoption of Protective Behaviors, and Frequency of Potential Coping Strategies**

**a) Risk Perceptions	b) Adoption of Protective Behaviors**

**c) Frequency of Potential Coping Strategies: Days in the Past Week when Someone…**

Panel a) displays the estimated marginal effects of Extraversion on risk perceptions for each period, based on OLS regressions of risk perceptions (0–100%) on period indicators, standardized extraversion, and their interaction. Each point represents the change in predicted risk perception (in percentage points) associated with a one–standard-deviation increase in extraversion.

Panel b) displays the estimated marginal effects of Extraversion on the likelihood of adopting a protective behavior for each period, based on logistic regressions of an adoption indicator on period indicators, standardized extraversion, and their interaction. Each point represents the change in the predicted probability (in percentage points) of adoption associated with a one–standard-deviation increase in extraversion. Green estimates exclude risk perceptions as a control; orange estimates condition on risk perceptions.

Panel c) displays the estimated marginal effects of Extraversion on the frequency of a potential coping strategy (days 0–7) for each period, based on OLS regressions including period indicators, standardized extraversion, and their interaction.

All regressions control for gender, race, age, education, marital status, household income, and the other personality traits. Horizontal bars denote 95% confidence intervals based on standard errors clustered at the individual level.

**Figure A.6: Marginal Effects of Openness on Risk Perceptions, Adoption of Protective Behaviors, and Frequency of Potential Coping Strategies**

**a) Risk Perceptions	b) Adoption of Protective Behaviors**

**c) Frequency of Potential Coping Strategies: Days in the Past Week when Someone…**

Panel a) displays the estimated marginal effects of openness on risk perceptions for each period, based on OLS regressions of risk perceptions (0–100%) on period indicators, standardized openness, and their interaction. Each point represents the change in predicted risk perception (in percentage points) associated with a one–standard-deviation increase in openness.

Panel b) displays the estimated marginal effects of openness on the likelihood of adopting a protective behavior for each period, based on logistic regressions of an adoption indicator on period indicators, standardized openness, and their interaction. Each point represents the change in the predicted probability (in percentage points) of adoption associated with a one–standard-deviation increase in openness. Green estimates exclude risk perceptions as a control; orange estimates condition on risk perceptions.

Panel c) displays the estimated marginal effects of openness on the frequency of a potential coping strategy (days 0–7) for each period, based on OLS regressions including period indicators, standardized openness, and their interaction.

All regressions control for gender, race, age, education, marital status, household income, and the other personality traits. Horizontal bars denote 95% confidence intervals based on standard errors clustered at the individual level.

**Figure A.7: Marginal Effects of Agreeableness on Risk Perceptions, Adoption of Protective Behaviors, and Frequency of Potential Coping Strategies**

**a) Risk Perceptions	b) Adoption of Protective Behaviors**

**c) Frequency of Potential Coping Strategies: Days in the Past Week when Someone…**

Panel a) displays the estimated marginal effects of agreeableness on risk perceptions for each period, based on OLS regressions of risk perceptions (0–100%) on period indicators, standardized agreeableness, and their interaction. Each point represents the change in predicted risk perception (in percentage points) associated with a one–standard-deviation increase in agreeableness.

Panel b) displays the estimated marginal effects of agreeableness on the likelihood of adopting a protective behavior for each period, based on logistic regressions of an adoption indicator on period indicators, standardized agreeableness, and their interaction. Each point represents the change in the predicted probability (in percentage points) of adoption associated with a one–standard-deviation increase in agreeableness. Green estimates exclude risk perceptions as a control; orange estimates condition on risk perceptions.

Panel c) displays the estimated marginal effects of agreeableness on the frequency of a potential coping strategy (days 0–7) for each period, based on OLS regressions including period indicators, standardized agreeableness, and their interaction.

All regressions control for gender, race, age, education, marital status, household income, and the other personality traits. Horizontal bars denote 95% confidence intervals based on standard errors clustered at the individual level.

**Figure A.8: Marginal Effects of Conscientiousness on Risk Perceptions, Adoption of Protective Behaviors, and Frequency of Potential Coping Strategies**

**a) Risk Perceptions	b) Adoption of Protective Behaviors**

**c) Frequency of Potential Coping Strategies**

Panel a) displays the estimated marginal effects of conscientiousness on risk perceptions for each period, based on OLS regressions of risk perceptions (0–100%) on period indicators, standardized conscientiousness, and their interaction. Each point represents the change in predicted risk perception (in percentage points) associated with a one–standard-deviation increase in conscientiousness.

Panel b) displays the estimated marginal effects of conscientiousness on the likelihood of adopting a protective behavior for each period, based on logistic regressions of an adoption indicator on period indicators, standardized conscientiousness, and their interaction. Each point represents the change in the predicted probability (in percentage points) of adoption associated with a one–standard-deviation increase in conscientiousness. Green estimates exclude risk perceptions as a control; orange estimates condition on risk perceptions.

Panel c) displays the estimated marginal effects of conscientiousness on the frequency of a potential coping strategy (days 0–7) for each period, based on OLS regressions including period indicators, standardized conscientiousness, and their interaction.

All regressions control for gender, race, age, education, marital status, household income, and the other personality traits. Horizontal bars denote 95% confidence intervals based on standard errors clustered at the individual level.
